# Beyond standard audiometry: Auditory dysfunction and steroid treatment outcomes in young adults following acoustic trauma

**DOI:** 10.1007/s00405-025-09989-3

**Published:** 2026-02-23

**Authors:** Yael Zaltz, Yael Solomon, Yisgav Shapira, Amit Wolfovitz, Daphne Ari-Even Roth, Yael Henkin

**Affiliations:** 1 Hearing, Speech, and Language Center, Sheba Medical Center, Tel Hashomer, Israel; 2 Department of Communication Disorders, Steyer School of Health Professions, Gray Faculty of Medical & Health Sciences, Tel Aviv University, Tel Aviv, Israel; 3 Department of Otolaryngology-Head and Neck Surgery, Sheba Medical Center, Tel HaShomer, Israel; 4 Gray Faculty of Medical & Health Sciences, Tel Aviv University, Tel Aviv, Israel

**Keywords:** Blast injury, UHF, DPOSE, Steroids, Acoustic trauma, Tinnitus

## Abstract

**Purpose:**

The impact of military noise exposure on auditory function is not well established and may not be fully captured by standard audiometry. This study aimed to evaluate: (1) the effects of military noise exposure on auditory function in young adults using (a) self-reported otological symptoms, (b) standard and ultra-high frequency (UHF; 9–14 kHz) audiometry, (c) distortion product otoacoustic emissions (DPOAE); (2) oral steroids effects on audiometric results, outer hair-cell function, and otological symptoms.

**Methods:**

A retrospective review of medical records was conducted for 189 young adults exposed to military noise. Participants with normal hearing underwent one assessment, while those with hearing loss or otological symptoms were reassessed after ~ 10 days and 3 months. Steroid-treated patients were compared to untreated controls.

**Results:**

Over half the cohort showed hearing loss in the standard or UHF range. Otological symptoms, particularly tinnitus and aural fullness, were prevalent even among individuals with normal standard-range thresholds. UHF audiometry and DPOAEs detected abnormalities missed by standard audiometry. Partial spontaneous recovery occurred within 10 days post-injury. Steroid treatment was linked to greater hearing thresholds improvements, particularly in the high-frequency range. UHF recovery was significantly greater in the right ear, suggesting asymmetry in vulnerability and recovery patterns.

**Conclusion:**

Military noise exposure leads to a highly prevalent and multifaceted impact on auditory function. Although treated patients showed greater gains, post-exposure threshold differences between treated and untreated patients restrict direct inference regarding steroid efficacy. Comprehensive assessment, including symptoms, UHF audiometry, and DPOAEs, is essential for accurate diagnosis and monitoring.

**Supplementary Information:**

The online version contains supplementary material available at 10.1007/s00405-025-09989-3.

## Background

Military noise exposure includes both continuous environmental noise and high-intensity, intermittent noise from weapons and explosions during combat, each posing a substantial risk for noise-induced hearing loss (NIHL) and other forms of auditory damage. Impulse noise, typically generated by firearms, consists of short-duration, high-intensity acoustic events that can reach 150–160 dB SPL and cause acoustic trauma, characterized primarily by cochlear mechanical and metabolic damage contributing to NIHL [1]. Animal studies show structural damage to the cochlea, including stereocilia disruption and nerve fiber swelling [2–4]. Human data reveal that acute acoustic trauma often affects hair cell rootlets, leading to cell death and neural degeneration [5]. Differently, blast injury results from an explosive shock wave that includes both acoustic and overpressure components, generating a broader spectrum of damage. Beyond cochlear effects, blast exposure may cause tympanic-membrane (TM) perforation, ossicular disruption, and additional neural or vestibular pathology [6–9]. While TM perforations often heal, ossicular and inner ear damage can result in lasting deficits [9]. Blast injuries can cause conductive, sensorineural, or mixed hearing loss [10].

Although traditionally military noise exposure is associated with a “noise-notch” audiogram peaking at 3–6 kHz, recent studies report greater losses at 4, 6, and 8 kHz, with 8 kHz often equal to or worse than 4 kHz [11]. It can also cause temporary threshold shift (TTS), which typically recovers within 30 days; beyond this point, the loss is considered permanent (PTS) [12]. Both TTS and PTS mainly affect high frequencies, and repeated TTS may lead to cumulative, irreversible damage. Notably, military noise exposure may lead to “hidden hearing loss”, auditory processing difficulties despite normal thresholds up to 8 kHz – affecting speech-in-noise comprehension and increasing listening effort [13–15]. This has been linked to cochlear synaptopathy [16–18]. Ultra-high frequency (UHF) audiometry has been proposed as a tool for detecting hidden loss [19]. Supporting this, emerging evidence reports UHF deficits, particularly between 11 and 14 kHz, after military noise exposure from firearms and other impulse sources [20, 21]. Moreover, recent findings suggest that UHF loss may also predict accelerated decline in the conventional (0.25–8 kHz) audiometric range [22].

Otoacoustic emissions (OAEs) offer a non-invasive method to detect cochlear damage in individuals with normal audiograms. Generated by outer hair cells (OHCs), OAEs are often reduced or absent when OHC function is compromised—frequently preceding threshold shifts [23, 24]. Since OHCs are especially vulnerable to noise-related and blast-related injuries, OAEs may serve as early markers of subclinical damage [7]. For example, in a large cohort study, significantly more absent DPOAEs were found among individuals with prior industrial or military noise exposure compared to non-exposed controls, despite normal hearing thresholds [25]. Furthermore, immediate reductions in DPOAE signal-to-noise ratios among aircrews were already observed following a single flight-mission noise exposure [21]. Longitudinal data further demonstrated that reduced DPOAE levels in military pilots with normal audiograms predicted subsequent hearing decline [26].

Beyond impairing communication, safety, and quality of life, military noise exposure, including but not limited to blast events, can lead to otological symptoms such as vertigo, hyperacusis, and tinnitus.

 [27–29]. Tinnitus, defined as the perception of sound without an external source, has been the most commonly recognized service-connected disability among U.S. veterans since 2006 [1]. Furthermore, tinnitus is also frequently linked to mental health conditions, including anxiety, depression, and substance use [27].

Recent studies have explored treatments for blast-related and impulse-noise–related hearing loss, including vasodilators, antioxidants, steroids, and hyperbaric oxygen therapy (HBOT) [30, 31]. Corticosteroids and HBOT are the most commonly used, mirroring approaches for sudden sensorineural hearing loss (SSNHL) due to their anti-inflammatory and oxygenation effects [31, 32]. However, clinical guidelines remain lacking due to variability in protocols and outcomes [31, 32]. A recent meta-analysis found mean hearing improvements of 6.55–9.02 dB with steroids alone and 7–12.41 dB with combined steroid-HBOT treatment, though results varied [31]. Notably, no studies evaluated effects beyond conventional audiometry (0.25–8 kHz) or included DPOAEs or UHF testing to assess outer hair cell or synaptic function.

Overall, despite evidence that noise exposure can damage UHF hearing and inner ear function, most NIHL research in military populations has focused on standard audiometry (0.25–8 kHz). Studies examining UHF audiometry and OAEs remain limited and inconsistent [20, 21], and the efficacy of steroid treatment is still unclear [31]. To address these gaps, the present study combined self-reports with comprehensive audiological and otological assessments, using a military-specific protocol with follow-up. It also compared outcomes between steroid-treated and untreated patients, offering new insights into NIHL diagnosis and management.

## Methods

### Study’s cohort

A retrospective analysis of data from 189 young adults who were referred to the Otolaryngology Department and the Hearing, Speech & Language Department at the Sheba Medical Center following exposure to acoustic trauma, including blast injury. All participants were treated uniformly according to institutional and Otolaryngology Society Guidelines.

The final cohort included 175 young adults (170 males), aged 18–30 years (M = 23.18 ± 3.0), with no prior hearing or otological history. This age range was selected to reduce the confounding effects of age-related hearing loss. Fourteen individuals were excluded due to inconsistent baseline audiograms.

### Audiological test battery

Participants underwent a comprehensive evaluation comprising:

1. **Questionnaire**

Participants completed a health and auditory status questionnaire (Appendix 1) covering physical and mental health, injury history, and hearing-related symptoms (e.g., hearing loss, tinnitus, aural fullness, hyperacusis, and difficulty understanding speech in noise).

2. **Standard (250–8000 Hz) and UHF (9**,**000–14**,**000 Hz) pure-tone audiometry**

Testing was conducted using the GSI AUDIOSTAR PRO audiometer. Normal hearing was defined as air-conduction thresholds ranging between 0 and 20 dB HL across octave frequencies (250 Hz–14 kHz). Hearing loss was defined as ≥ 2 thresholds ≥ 25 dB HL or ≥ 1 threshold ≥ 30 dB HL.

3. **DPOAE**

DPOAEs were recorded in a soundproof room using an Otoport Advance system (Otodynamics Ltd, UK) with a UGD probe. Stimuli were paired primary tones (f1/f2, ratio 1.22) at 65/55 dB SPL. Frequencies tested: 2000, 3000, 4000, 6000, and 8000 Hz. For each frequency, the emission level and noise floor were recorded, with noise estimated as two standard deviations above the mean. Responses were considered present if emission amplitude exceeded 0 dB SPL with SNR ≥ 6 dB. The > 0 dB SPL criterion was applied to ensure inclusion of only robust emissions clearly above the noise floor. Responses were classified as abnormal if absent at ≥ 2 frequencies, including one within the 2–4 kHz range.

4. **Tympanometry**

In cases with suspected middle-ear involvement, 226 Hz probe-tone tympanometry was also conducted, using Interacoustics Titan Tympanometer.

## Procedure

The first audiological evaluation was conducted as early as possible, with most participants assessed within three weeks post-injury. A smaller subset underwent later evaluations, typically due to medical or logistical constraints. Patients exhibiting hearing loss were referred to an otolaryngologist for microscopic otoscopy and assessment for steroid treatment. Oral prednisone (60 mg/day for 7 days) was prescribed only if initiated within 14 days post-exposure, based on (1) confirmed sensorineural hearing loss (≥ 30 dB HL at one frequency or ≥ 25 dB HL at two or more frequencies within 250–8000 Hz) or (2) physician discretion (e.g., abnormal DPOAEs, blast injury with TM perforation, or persistent symptoms, including hearing loss, aural fullness, or new-onset tinnitus > 24 h). All patients with hearing loss underwent follow-up audiological evaluations 10 days and 3–4 months after the initial assessment, in line with recovery timelines following acoustic trauma [12].

Ethics approval was obtained from the Sheba Medical Center’s (SMC) Institutional Review Board (reference number: 1534-14 SMC) and included full exemption from informed consent as the utilized methods included routine clinical tests used at the SMC. All tests were performed in accordance with the relevant guidelines and regulations.

## Statistical analysis

Two mixed-effects linear models were fit, using SAS PROC MIXED to evaluate changes in hearing thresholds between the first and second assessments. In the first model, the dependent variable was the mean threshold (in dB). Fixed effects included group (steroid-treated vs. untreated; defined as CLASS), threshold range (PTA: pure-tone average at 0.5–2 kHz; HF PTA: 3–8 kHz; UHF PTA: 11.2–14 kHz), assessment number (1 vs. 2), and ear (right vs. left). This model assessed the main effects and interactions of these factors on hearing thresholds while accounting for individual variability. The second model examined the change in thresholds (Δ dB) between assessments as the dependent variable, with the same fixed effects except for assessment number.

Chi-square tests were used to assess whether the proportion of self-reported otological symptoms and the prevalence of absent DPOAE responses (in frequencies with normal hearing) changed significantly between the two assessments.

## Results

The first evaluation was conducted, on average, 23.26 (± 15.81) days post-injury, with a median of 20.5 days (range: 1–73). Most evaluations occurred within the first 21 days (89 participants), while evaluations beyond this period contributed to the broad variability observed. Otological symptoms were reported at the participant level, with 54% of participants (95/175) reporting at least one symptom. The most prevalent symptoms were tinnitus (36%), aural fullness (27%), hyperacusis (11%), and difficulty understanding speech in noise (5%). Other otological symptoms (18%) were dizziness, vertigo, aural pressure, and pain. Notably, 27% of the entire cohort reported tinnitus in combination with at least one other otological symptom.

Hearing status, examined per participant, revealed that 56% of the cohort (98/175) exhibited hearing loss in the standard audiometric range, UHF range, or both. Among these, 55% had unilateral hearing loss (30 right ear, 24 left ear). Hearing loss (≥ 25 dB HL) limited to the UHF range was observed in 11% of the cohort (n = 19). Among participants with hearing loss, tinnitus (32%) and aural fullness (26%) were most common. Notably, among the 77 individuals with normal hearing in the standard range, 30% (n = 23) reported symptoms – 13 of whom had UHF-only hearing loss.

Because audiometric configuration and DPOAE results are ear-specific, the following analyses are reported per ear. Of the 116 ears with hearing loss in the standard frequency range, 96 (83%) exhibited sensorineural hearing loss, while the remaining demonstrated conductive or mixed loss, including cases with TM perforation (n = 7). Severity distributions for standard and UHF ranges are presented in Table 1.

**Table 1:** The prevalence of hearing loss severity in the standard and UHF frequency ranges

Hearing loss severity (dBHL)	PTA (0.5–2 kHz) (*N* = 349 ears)	HF PTA (3–8 kHz) (*N* = 349 ears)	UHF (11.2–14 kHz) (*N* = 324 ears)
Normal Hearing (0–20)	303 (86.8%)	260 (74.5%)	222 (68.5%)
Mild HL (21–40)	38 (10.9%)	61 (17.5%)	62 (19.1%)
Moderate HL (41–55)	5 (1.4%)	15 (4.3%)	18 (5.6%)
Moderate to Severe HL (56–70)	0 (0%)	8 (2.3%)	14 (4.3%)
Severe to Profound HL (≥ 71)	3 (0.9%)	5 (1.4%)	8 (2.45%)

**Fig. 1:**
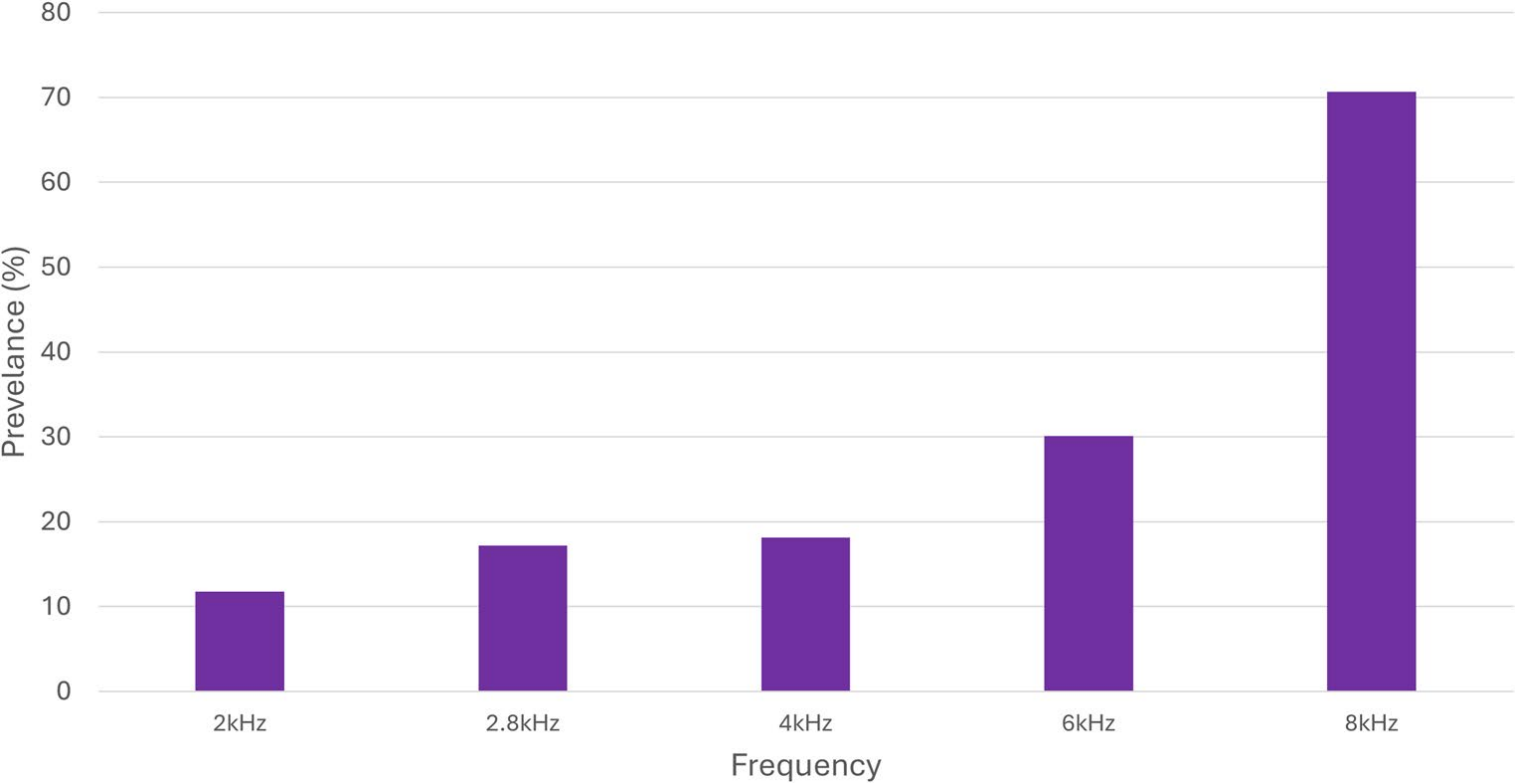
The prevalence of absent DPOAE responses in each frequency, among ears with normal hearing thresholds (≤ 20dB HL) in the standard frequencies

DPOAE responses were abnormal in 27% (46/172) of ears with normal thresholds in the standard frequency range and no evidence of middle ear pathology. Notably, among patients with normal thresholds in the standard frequency range but with otological symptoms (*n* = 23), 52% *of the ears* showed abnormal DPOAEs. Figure 1 displays the prevalence of absent DPOAE responses by frequency in ears with normal hearing thresholds in the standard frequencies.

### Second evaluation

Sixty-one patients (mean age 23.84 ± 3.28 years) met the inclusion criteria for the second evaluation, conducted on average 11.72 (± 5.28) days (median = 10.0 days) after the first. Of these, 27 (44%) patients received steroid treatment. Otological symptoms remained largely unchanged between evaluations in both groups (*p* >.3; see Table 2).

**Table 2:** Otological symptoms – first and second evaluation (By Group)

	*Steroidal Treatment* (*N* = 27)		*No Treatment* (*N* = 34)	
	1 st Evaluation	2nd Evaluation	1 st Evaluation	2nd Evaluation
Tinnitus	21 (77.8%)	18 (66.7%)	11 (32.4%)	11 (32.4%)
Fullness	10 (37%)	11 (40.7%)	9 (26.5%)	9 (26.5%)
Hyperacusis	4 (14.8%)	4 (14.8%)	4 (11.8%)	5 (14.7%)
Speech Understanding Difficulties	0 (0%)	0 (0%)	4 (11.8%)	4 (11.8%)
Other Otological Symptoms	7 (25.9%)	4 (14.8%)	9 (26.5%)	8 (23.5%)

**Fig. 2:**
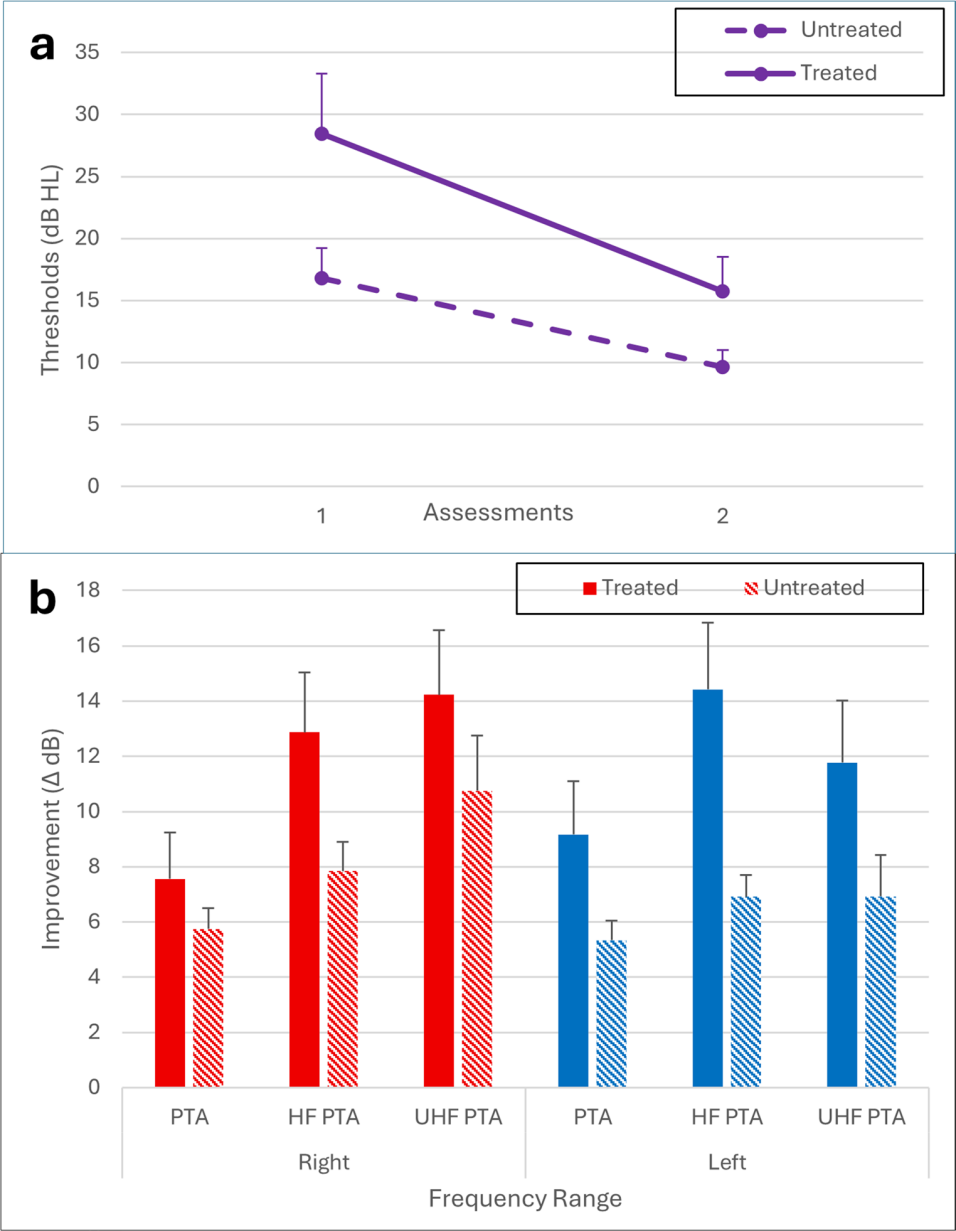
(**a**) Mean (+ 1 SE) hearing thresholds (dB HL) for the treated and untreated groups across the first and second assessments. (**b**) Threshold improvement (∆ dB) between the first and second assessments for each group, shown separately for PTA, HF PTA, and UHF PTA ranges. PTA = 0.5–2 kHz, HF PTA = 3–8 kHz, UHF PTA = 11.2–14 kHz

59% of the cohort (*n* = 36) exhibited hearing loss in the second evaluation, in the standard audiometric range, UHF range, or both. Among these, 69.44% presented unilateral hearing loss (15 right ear, 10 left ear). Hearing loss (≥ 25 dB HL) limited to the UHF range was observed in 21.31% (*n* = 13) of the patients. Analysis revealed significant main effects for group [*F(1*,* 59) = 12.11* *p* *=.0009*] and assessment [*F(1*,* 59) = 108.46* *p* *<.0001*], as well as significant group X assessment interaction [*F(1*,* 59) = 7.26* *p* *=.0092*]. This interaction indicates that although the untreated group had better thresholds overall (*p* *=.0012*) and both groups showed significant improvement between the first and second assessments (*p* *<.0001*), the improvement was significantly larger in the treated group (Fig. 2a). Significant main effects were also found for frequency range [*F(2*,* 112) = 42.58* *p* *<.0001*], with significant group X frequency range interaction [*F(2*,* 112) = 7.51* *p* *=.0009*], indicating that the difference in thresholds between the treated and untreated groups was larger in the HF and UHF ranges. A significant group X ear interaction [*F(1*,* 58) = 5.81*,*p* =.0191] indicated that the difference in thresholds between the groups was larger in the left ear. No other interactions have reached significance (*p* *>.05*).

Threshold improvements between the first and second assessments (∆ dB) are presented in Fig. 2b. Analysis revealed significant main effects of group [*F*(1, 59.2) = 4.58, *p* =.0365] and frequency range [*F*(2, 55.4) = 5.59, *p* =.0061], as well as a significant range X ear interaction [*F*(2, 53.7) = 4.96, *p* =.0105] and near-significant frequency range X group interaction [*F*(2, 55.4) = 3.05, *p* =.055]. Follow-up analyses revealed no significant differences between groups and ears in ∆ dB within the PTA range (*p* *>.05*). However, in the HF PTA range, a significant main effect of group was observed, with the steroid-treated group showing larger improvements than the untreated group [*F*(1, 59) = 5.77, *p* =.0195]. In the UHF PTA range, a significant main effect of ear emerged, indicating better recovery in the right compared to the left ear [*F*(1, 51) = 9.48, *p* =.0033]. No other main effects or interactions have reached significance (*p* *>.05*).

DPOAE responses in the second assessment were abnormal in 31.9% (22/29) of the ears that exhibited hearing within the normal range in the standard frequencies, including 19.2% in the treated group and 39.5% in the untreated group. This difference did not reach statistical significance (*p =.78*). Furthermore, no significant differences in DPOAE presence were observed between the first and second evaluations in either group (*p* *>.093*).

### Final evaluation

Thirty patients (all male) met inclusion criteria for the final evaluation, conducted 3–4 months post-injury (mean 113.9 ± 33.2 days). Otological symptoms were reported by 73% (22/30), with tinnitus being most common (95%). Hearing loss was present in 50% (*n* = 15), 40% of whom had unilateral loss (2 right, 4 left). An additional 13.3% (*n* = 4) had UHF-only loss (≥ 25 dB HL). Mean thresholds were: PTA = 17.14 dB (range: 0–120), HF PTA = 22.21 dB (range: 0–117.5), and UHF PTA = 20.59 dB (range: 0–120). DPOAE responses were abnormal in 36.1% (13/36) of ears that exhibited hearing within the normal range in the standard frequencies.

## Discussion

The key findings of the present study indicate that: (1) hearing loss *is highly prevalent following military noise exposure*, whether within the standard audiometric range, the UHF range, or both. More than half of the cohort exhibited some degree of auditory threshold elevation. 2) Otological symptoms, particularly tinnitus and aural fullness, were frequently reported. Notably, nearly one-third of participants with normal hearing thresholds in the standard audiometric range experience these symptoms, underscoring the insufficiency of conventional audiometry in detecting all cases of functional auditory impairments. (3) UHF audiometry and DPOAE testing uncovered pathological changes even among participants with normal thresholds in the standard frequency range. These findings emphasize the diagnostic value of extending audiological evaluation beyond standard frequencies thresholds. (4) A high degree of spontaneous improvement in hearing thresholds, across the entire frequency range, was noticed at the second assessment, performed approximately 10 days after the initial evaluation (5) Although steroid therapy was associated with greater threshold gains, significant benefits were observed only in the HF PTA range. (5) Interestingly, recovery in the UHF range was significantly greater in the right ear compared to the left, suggesting potential lateralized vulnerability or differential recovery patterns. Taken together, these findings highlight the limitations of standard audiometry in capturing the auditory consequences of acoustic trauma including blast injuries during military service. They also reinforce the clinical utility of incorporating UHF audiometry and DPOAE into post-injury hearing assessments. While partial recovery may occur spontaneously or be aided by medical intervention, the overall benefit of steroid treatment appears modest and limited to specific frequency ranges.

### The impact of military noise exposure

The findings from the first evaluation, conducted within three weeks post-exposure, expand and reinforce previous findings on auditory impairments following *military noise exposure*. Consistent with prior studies [10], high-intensity military-related noise was associated with a range of auditory deficits, including varying degrees of hearing loss and otological symptoms. Nearly half (45%) of the examined patients exhibited hearing loss in at least one ear at standard audiometric frequencies (0.25–8 kHz), comparable to prior data (e.g., 49% [33]). Most cases were sensorineural, with the most pronounced deficits observed in the high-frequency range (3–8 kHz), supporting evidence that military acoustic trauma often extends into higher frequencies rather than presenting a classic noise-notch pattern [11].

Importantly, our findings highlight the clinical value of UHF audiometry, as 11% of patients with otherwise normal hearing exhibited deficits exclusively in the 9–14 kHz range. These individuals reported a higher prevalence of otological symptoms, including tinnitus, hyperacusis, and aural fullness, than those with normal UHF hearing, supporting the role of UHF testing in identifying early cochlear damage [20, 21]. By providing an audiological correlate for otherwise unexplained otological symptoms, UHF audiometry improves the ability to link subjective symptoms with measurable auditory dysfunction, reinforcing its clinical relevance in evaluating military NIHL. Given that noise-induced cochlear synaptopathy, marked by disrupted synapses between inner hair cells and auditory nerve fibers and has been linked to hidden hearing loss [16, 17], the increased prevalence of symptoms (tinnitus and aural fullness) in patients with isolated UHF deficits suggest these symptoms may reflect early-stage cochlear pathology *related to military noise exposure*.

An additional finding that underscores the limitations of behavioral audiometry is that absent DPOAE responses despite normal audiograms were evident in 13–18% of the ears at 2–4 kHz, increasing to 30% at 6 kHz and 70% at 8 kHz. This pattern suggests early outer hair-cell dysfunction that precedes measurable threshold shifts. A similar frequency-dependent trend was reported by Attias et al. [25], who found a progressive increase in the incidence of absent DPOAEs, from 9% at 2 kHz to 52% at 6 kHz, in a large cohort of individuals with a history of industrial or military noise exposure, despite normal audiograms. This aligns with previous research suggesting that DPOAEs can serve as early biomarkers of NIHL, identifying individuals at risk for progressive loss before audiometric thresholds are affected [21, 26]. It should be noted that technical challenges, such as standing waves in the ear canal and probe calibration challenges, can impair stimulus delivery and reduce the reliability of high-frequency DPOAE measurements [34]. TogetherTogetherTogether, these findings reinforce the utility of DPOAEs in detecting subclinical cochlear changes that are not captured by pure-tone thresholds alone and highlight the need to interpret high-frequency DPOAE results cautiously, considering technical factors.

Tinnitus was the most frequently reported symptom, affecting over one-third of participants, consistent with previous reports identifying it as a common service-related disability among military personnel [27, 29]. More broadly, otological symptoms, including tinnitus, aural fullness and hyperacusis, were more prevalent among participants with hearing loss, whether in the standard audiometric range or restricted to UHF, than in those with normal hearing. This further underscores the limitations of standard audiometry, as auditory symptoms may reflect underlying cochlear dysfunction undetectable by standard testing.

Taken together, these findings underscore the need for comprehensive audiological evaluations following military noise exposure, incorporating measures such as DPOAEs and UHF audiometry. Moreover, early cochlear damage has been associated with tinnitus, hyperacusis, difficulties in speech-in-noise perception, and increased listening effort in challenging listening conditions [15, 18]. It may also indicate a heightened risk of progressive hearing loss [22], making the integration of these tools essential for accurate detection and monitoring of auditory impairments in military personnel.

### The effect of oral steroid treatment

#### Steroid-treated group

Significant improvements in hearing thresholds in patients treated with steroids amounted to 8 dB (± 9.3) in the PTA range and 13.6 dB (± 11.8) in the HF PTA range. These results align with a recent study evaluating the effects of a similar steroid regimen in military personnel with acute acoustic trauma [35], and somewhat exceed those reported in a recent meta-analysis of military NIHL treatments [31]. Most importantly, this is the first study to evaluate recovery in the UHF range (9–14 kHz), revealing a significant 13 ± 10.9 dB improvement following steroid treatment. Given that high-frequency regions are particularly susceptible to noise-induced damage [11], these results underscore the value of including UHF audiometry, alongside standard audiometry, in both diagnosis and treatment.

Importantly, the steroid-induced improvements observed here, though significant, are notably smaller than those typically reported for SSNHL [36], likely reflecting underlying pathophysiological differences. SSNHL is often attributed to reversible mechanisms such as infectious diseases, and vascular and hematological disorders that typically respond well to corticosteroids [37]. In contrast, acoustic trauma *and* blast injur*ies* primarily cause mechanical damage to cochlear structures, including hair cells, stereocilia, and synapses [4]. However, it also triggers secondary metabolic processes such as oxidative stress, excitotoxicity, and inflammatory responses [4, 38]. While steroids are unlikely to reverse structural damage, they may help mitigate these secondary effects, contributing to partial functional recovery.

Taken together, this suggests that steroid treatment may aid recovery in high frequencies by reducing inflammation, improving cochlear blood flow, and mitigating oxidative stress. However, despite these improvements, hearing thresholds did not fully normalize in most steroid-treated cases, suggesting that while corticosteroids may support partial recovery, they are insufficient to fully restore cochlear function following noise-induced damage.

Despite the above-described improvements in hearing thresholds, no significant changes were observed in otological symptoms, including tinnitus, aural fullness, and hyperacusis. This is consistent with mixed findings in the literature regarding steroid efficacy for subjective symptoms. While Van Haesendonck et al. [39] reported reductions in tinnitus severity following steroid treatment (with or without HBOT), other studies have documented persistent or worsening symptoms regardless of treatment. For example, long-term symptoms such as tinnitus, hyperacusis, and difficulty hearing in noise were reported after the Boston Marathon bombing, independent of steroid use [7].

The persistence of subjective symptoms despite audiometric recovery may reflect several underlying mechanisms. First, while steroid treatment may restore some cochlear function, lasting changes such as deteriorated synaptic connections between inner hair cells and auditory nerve fibers – i.e., cochlear synaptopathy – may continue to drive symptoms like tinnitus and hyperacusis [18]. Given that synaptopathy can disrupt neural signaling without affecting audiometric thresholds [16, 17], steroid treatment may not address these deficits. Moreover, subjective symptoms may involve central mechanisms, including alterations in the auditory cortex, limbic system, or brainstem that lie beyond the reach of peripheral-focused therapies [40].

#### Untreated group

The magnitude of improvement was consistently lower across all frequency ranges in the no-treatment compared to treatment group, although statistically significant differences between groups were limited to the HF range. These modest gains align with prior reports of spontaneous recovery after acoustic trauma, reflecting TTS in the early post-exposure period [12]. As in the treated group, no significant changes were observed in DPOAE responses or otological symptoms, suggesting that underlying cochlear or neural dysfunction persisted despite partial threshold recovery.

It is important to note that steroid treatment was not withheld arbitrarily. In cases with mild symptoms or minimal audiologic involvement, some patients opted to forgo steroid therapy due to operational demands of military service, where steroid use may temporarily restrict active-duty participation. This consideration, combined with the lower clinical severity in these cases, influenced the decision not to initiate treatment. As a result, the untreated group had more favorable baseline hearing thresholds and a higher likelihood of spontaneous recovery, consistent with existing evidence that milder forms of hearing loss are associated with faster and more complete recovery [37]. Therefore, the untreated group cannot be considered a true control for the steroid-treated group. This baseline imbalance reflects confounding by indication and should be taken into account when interpreting treatment outcomes. Notably, a similar trend was observed in the only other study comparing treated and untreated military personnel with acute acoustic trauma, which likewise reported better baseline thresholds and smaller improvements in the untreated group [35].

An interesting finding of the present study was the asymmetry in hearing recovery, with greater improvement in UHF thresholds in the right ear, regardless of treatment. This aligns with the well-documented “right ear advantage” in NIHL recovery, where the left ear is more susceptible to high-frequency damage and shows less recovery [41, 42]. For example, in cases of NIHL with a unilateral 2 kHz notch, the left ear was reported to exhibit poorer threshold in over 80% of patients – indicating greater left-ear susceptibility to noise injury [43]. Similarly, in a large-scale study of 538 adolescent boys with early-stage NIHL, significantly poorer high-frequency thresholds were observed in the left ear compared to the right [44]. Other studies have shown greater threshold shifts and slower recovery in the left ear [42]. Although the precise mechanisms underlying this right ear advantage remain under investigation, the current results support this asymmetry and highlight the need to consider interaural differences in both diagnosis and auditory rehabilitation.

## Limitations

While this study provides novel insights into the effects of military noise exposure on auditory function, several limitations should be considered. First, the number of participants in the second and, more so, the third follow-up was relatively small, limiting the ability to determine whether improvements in hearing thresholds were sustained or if additional changes emerged over time. Long-term follow-up studies with larger groups are needed to better characterize and clarify the trajectory of auditory recovery. Second, while UHF audiometry and DPOAE testing provided valuable insights into cochlear function, functional hearing assessments such as speech-in-noise testing were not included. Given that many military personnel experience auditory difficulties in complex environments despite normal audiometric thresholds, incorporating such measures could offer a more comprehensive understanding of the consequences of military noise exposure. Finally, the difference in baseline hearing between the steroid-treated and untreated groups, while clinically justified, poses a challenge to making direct inferences regarding the efficacy of steroid treatment.

## Conclusions

The current findings indicate that the effect of military noise exposure in young adults extends beyond the conventional audiometric frequency range, with significant implications for clinical assessment and management. The high prevalence of otological symptoms, combined with findings of subclinical cochlear dysfunction manifested in DPOAE and UHF thresholds, underscore the importance of a comprehensive evaluation protocol to accurately diagnose and monitor hearing loss in military personnel, especially for individuals with persistent symptoms despite normal standard audiometry.

Post-exposure hearing-threshold differences between the steroid-treated and untreated groups restrict direct comparison of steroid efficacy compared to spontaneous recovery patterns. Nevertheless, current results indicate that the treated group exhibited partial recovery of hearing thresholds, particularly in the high frequency range. The persistence of symptoms such as tinnitus, aural fullness, and/or hyperacusis, despite audiometric improvement suggests that post-exposure hearing difficulties may extend beyond hair-cell damage, potentially involving deficits in central auditory processing. These findings align with previous research indicating that pharmacological interventions alone may be insufficient to mitigate the long-term effects of noise trauma. Overall, the study highlights the complex nature of military noise exposure in young adults and underscores the need for improved screening, monitoring, and treatment strategies tailored for this high-risk population. By incorporating more sensitive diagnostic measures, audiologists can more effectively assess the long-term functional impact of military noise exposure and deliver more personalized and effective auditory rehabilitation.

## Supplementary information

Below is the link to the electronic supplementary material.

Supplementary File 1 (DOCX 15.5 KB)

## Data Availability

The datasets used and analyzed during the current study are available from the corresponding author on reasonable request.
