# Supplementary material for: Beyond standard audiometry: Auditory dysfunction and steroid treatment outcomes in young adults following acoustic trauma
Source: Eur Arch Otorhinolaryngol. 2026 Feb 23;283(4):2329–38. doi: 10.1007/s00405-025-09989-3 (PMC13053575; doi:10.1007/s00405-025-09989-3)
Supplement: Supplementary file 1 — (DOCX 15.5 KB) [file 405_2025_9989_MOESM1_ESM.docx]

**Appendix 1. Self-report Questionnaire**

**Exposure and Hearing History**

1. How much time has passed since the incident?
2. Were you exposed to gunfire? If yes, please specify the type of weapon, the duration of exposure, and the context.
3. Did you use hearing protection during the exposure?
4. Were you exposed to a blast, or did you sustain a blast-related injury?
5. Have you experienced bleeding or any fluid coming from the ears?
6. A. Did you notice any change in your hearing during or after the incident? If so, when did it occur?
   B. Since the initial change, has your hearing further improved or worsened?
7. Did you experience a loss of consciousness during the event?
8. Were you confused or disoriented at any point?
9. Did you experience any memory disturbances following the event?
10. Had you been exposed to loud noise prior to this event?
11. Have you undergone a hearing evaluation in the past?

**Current Symptoms and Complaints**

1. Do you experience tinnitus (ringing or buzzing in the ears)?
2. Do you feel a sensation of fullness in your ears?
3. Do you experience ear pain?
4. Do you feel pressure in your ears?
5. Have you experienced dizziness or a sense of unsteadiness?
6. Are you unusually sensitive to loud sounds or certain voices?
7. Do voices or sounds appear distorted?
8. Do you have difficulty understanding speech in noisy or quiet environments?
